# Diseases of the Nervous System

**Published:** 1913-03

**Authors:** 


**Diseases of the Nervous System.** By JUDSON S. BURY, M.D. Lond., F.R.C.P. Pp. xx, 778. Manchester: At the University Press. 1912.

In this text-book the whole field of nervous diseases is covered within a moderate compass. The literature of the subject is now so great as to make it difficult to avoid excessive length, and yet leave out nothing essential. This problem has been fairly well met in the present volume. The book is well and clearly written, and the illustrations are numerous and well selected to elucidate the text. The coloured plates illustrating cerebral hemorrhage are especially good. As we should expect from the author's previous work, the section on multiple neuritis is particularly well done. That on intracranial tumours might be somewhat extended with advantage on account of the importance of accurate diagnosis in these cases, and also of their difficulty. We think that in the future editions, which we hope to see, that more might be said about the experimental pathology and epidemiology of anterior poliomyelitis, of the various clinical types of disseminated sclerosis, and of the subsidiary signs in hemiplegia, which aid in the distinction of a hemiplegia of organic from one of functional origin. The author retains ataxic paraplegia as a convenient clinical term, whilst being careful to point out that the large majority of cases come under either subacute combined degeneration of the cord or disseminated sclerosis. We think, however, that something more might be added as to the pathological changes in the cord in these cases, which seem to us to show important differences from those found in the more common group of cases associated with profound anæmia. Personally, we should put still more strongly than the author has done the importance in cerebral syphilis of the efficient use of mercurial inunctions, and also that as syphilis can now be excluded by modern tests the use of iodide of potassium in non-syphilitic cerebral tumours has only a limited application. These are but minor criticisms. The general plan and execution of the book is good, the type clear, and it is successful in giving an adequate presentment of the subject.

